# Increased plasma lipoprotein-associated phospholipase A2 levels are associated with coronary slow flow

**DOI:** 10.1186/s12872-020-01463-8

**Published:** 2020-05-27

**Authors:** Yao-dong Ding, Yu-qiang Pei, Rui Wang, Jia-xing Yang, Yin-xin Zhao, Xiao-li Liu, Hua Shen, Qian Ma, Shuo Zhang, Hai-long Ge

**Affiliations:** 1 Department of Cardiology, Beijing Anzhen Hospital, Capital Medical University, Beijing, 100029 PR China; 2 Beijing Tiantan Hospital, Capital Medical University, Beijing, China

**Keywords:** Coronary slow flow, Lipoprotein-associated phospholipase A2, Coronary artery disease

## Abstract

**Objective:**

Coronary slow flow (CSF) is characterized by delayed opacification of distal epicardial coronary arteries without significant coronary stenosis. In addition, The changes of lipoprotein-associated phospholipase A2 (Lp-PLA_2_) as a significant predictive factor for CSF remain controversial. The study aims to investigate the association between plasma Lp-PLA_2_ and CSF.

**Methods:**

In this retrospective study, 170 consecutive patients who underwent coronary angiography were enrolled in Beijing Anzhen Hospital from January 2017 to September 2019, and were divided into CSF group and normal control groups. According to coronary blood flow rate measured by the thrombolysis in myocardial infarction frame count (TFC) method, CSF was defined as TFC > 27. Serum Lp-PLA_2_ levels were measured in an enzyme-linked immunosorbent assay.

**Results:**

Lp-PLA_2_ levels were higher in the CSF group than in the control group (288.6 ± 50.3 versus 141.9 ± 49.7, *P* < 0.001) and were significantly correlated with the mean coronary artery thrombolysis in myocardial infarction (TIMI) frame count (r = 0.790, P<0.001). Logistic regression analysis showed that high Lp-PLA_2_ was independently associated with CSF after adjustment for conventional risk factors (OR = 1.040, CI = 1.022–1.059, P<0.001). Male sex (OR = 2.192, CI = 1.161–4.140, *P* = 0.016) and hypertension (OR = 1.965, CI = 1.034–3.736, *P* = 0.039) were also CSF risk factors. Receiver-operating characteristic curve (ROC) analysis showed that Lp-PLA_2_ levels can predict CSF severity; the predictive power was higher than the other risk factors.

**Conclusion:**

Our study demonstrated that patients with CSF had higher circulating levels of Lp-PLA_2_ than normal controls. After adjustment for potential confounders, increased Lp-PLA_2_ was independently associated with presence of CSF.

The coronary slow flow (CSF) phenomenon is a special phenomenon involving coronary microcirculation dysfunction, which is characterized by delayed distal vascular opacification without significant epicardial coronary stenosis. Studies have shown that slow blood flow is often associated with adverse cardiovascular events, including angina, myocardial infarction, malignant arrhythmias and even sudden cardiac death, suggesting a poor prognosis [1]. Although it has been 40 years since Tambe et al. first discovered slow blood flow [2], the exact mechanism of its pathogenesis remains unclear. A large number of studies have shown that the pathogenesis of CSF may involve vascular endothelial dysfunction, vascular inflammation, obesity, atherosclerosis and other factors [3–5]. However, there is a lack of effective biomarkers that specifically predict CSF. Recently, a vast majority of evidence has indicated that many inflammatory mediators such as interleukin-1, interleukin-10, and the lymphocyte to monocyte ratio, are associated with the pathogenesis of CSF, indicating the presence of a proinflammatory process occurring as a result of the phenomenon [6–8]. Thus, it is of value to identify the risk factors for CSF in order to detect and prevent CSF as early as possible.

Lipoprotein-associated phospholipase A2 (Lp-PLA_2_), a leukocyte-derived enzyme, is involved in the metabolism of low-density lipoprotein (LDL), propagating atherogenesis, and mediating the inflammatory process of the vascular wall [9]. Lp-PLA_2_ circulates in plasma in its active form with complexes of LDL and high density lipoprotein (HDL) [10]. Recent evidence suggests that Lp-PLA_2_ plays an important role in the pathophysiology of atherosclerosis and as a predictive biomarker for predicting future cardiovascular events [11]. According to its properties of proinflammation and atherogenesis, and the fact that increased Lp-PLA_2_ has been found to be closely associated with inflammation and atherosclerosis, we speculate that Lp-PLA_2_ may be associated with CSF [12]. Therefore, in this study, our aim was to evaluate the relationship between coronary blood flow and the Lp-PLA2 level.

## Methods

### Study patients

The retrospective study was carried out from January 2017 to September 2019 at Anzhen Hospital. A total of 170 patients who underwent coronary angiography with clinical chest pain and without significant coronary stenosis were consecutively enrolled in the observational study; 78 of the patients had slow coronary flow without any stenosis evidenced by coronary angiography, and 92 patients had normal coronary arteries and normal flow. Patients with prior evidence of coronary artery disease (acute coronary syndrome, coronary interventions history, coronary plaque and significant atherosclerotic lesions (stenosis above 40%), coronary ectasia, coronary calcification), congenital heart disease, valvular heart disease, cardiomyopathy, ischemic electrocardiogram, hematological system disease, tumors, heart failure, liver dysfunction, or kidney dysfunction or who had recently undergone surgery were excluded from the study. The study was approved by the Beijing An zhen Hospital Ethics Committee of Capital Medical University, and all patients provided informed consent.

### Anthropometric and laboratory measurements

The study measured demographic characteristics, including age, sex, height, weight, body mass index (BMI) and waist circumference. Smoking, hypertension, diabetes mellitus and medication use were collected from electronic medical records. After fasting for at least 8 h, peripheral blood was collected on the morning of the first day of admission and stored at − 70 °C. The plasma Lp-PLA_2_ activity was measured using an enzyme-linked immunoassay (PLAC™ test, DIADEXUS, USA). Glucose, urea, creatinine, total cholesterol (TC), triglyceride (TG), HDL, and LDL levels were measured using a chemiluminescence method with a Roche Diagnostics Cobas analyzer Cobas 8000, c702 module. High sensitivity C-reactive protein (hs-CRP) levels were also measured by the nephelometric method. All samples were tested in triplicate according to the manufacturer’s protocols.

### Diagnostic criteria

ACS was diagnosed according to European Society of Cardiology guidelines in 2015 [13]. Cardiomyopathy was diagnosed by established histological, immunological, and immunohistochemical criteria according to the WHO classification [14]*.* The diagnostic criteria of diabetes mellitus was according to WHO guidelines or by indication for insulin or anti-diabetic medications [15]. Hypertension was defined as repeated systolic pressure ≥ 140 mmHg and/or diastolic pressure ≥ 90 mmHg at least twice, or previously diagnosed hypertension [16].

### Coronary angiography protocols

CSF was defined according to the thrombolysis in myocardial infarction (TIMI) frame count (TFC) method. Coronary angiogram was performed using a digital subtraction angiography system (Allura Xper FD20; Philips Medical Systems, Best, the Netherlands), which used the standard Judkins technique to obtain images at the rate of 30 frames/s. The injection rate was 4–5 ml/s in the left coronary artery and 3–4 ml/s in the right coronary artery (RCA). The left coronary artery was injected with 8-10 ml contrast agent and RCA was injected with 6-8 ml contrast agent. The left anterior descending coronary artery (LAD) was imaged as the right anterior oblique projection with an angle of 20–25°, the left circumflex coronary artery (LCX) was imaged as the right anterior oblique projection with an angle of 20–25°, and the RCA was imaged as the positive projection with an angle of 30°. The first frame was defined when the contrast agent touched two medial walls of the coronary artery and advanced steadily with a diameter of more than 70%, and the last frame was defined when the leading edge of the contrast agent reached the end of the branches of the coronary artery. The frame counts in the LAD were divided by a factor of 1.7 to correct for its longer length. According to Gibson et al., any frame count over 27 is considered abnormal and indicates significant CSF [17]. All patients were carefully monitored for pulse and blood pressure during coronary angiogram.

### Statistical analysis

Data were analyzed with IBM SPSS software version 23.0. Categorical variables were presented as frequencies and percentages, and continuous variables were expressed as the mean and standard deviation. Continuous variables with a normal distribution were compared using Student’s t-test. Kruskal-Wallis test and one-way ANOVA were used to analyze the differences between groups. The categorical variables were tested by chi-square test. Spearman ρ test correlation analysis was used to examine the correlation between Lp-PLA_2_ and other risk factors. We used multivariate logistic regression analysis to assess the value of Lp-PLA_2_ in predicting the presence of CSF with adjustment for risk factors. Additionally, a receiver operating characteristic (ROC) curve was plotted for the plasma Lp-PLA2 level to evaluate the ability of the variable to classify the severity of CSF. The ROC curve were calculated in an area under the curve (AUC) and 95% confidence intervals. A *P* value< 0.05 (two tailed) was considered significant.

## Results

### Patient characteristics

The baseline clinical characteristics of the study participants are shown in Table 1. We enrolled 170 patients (mean age 61.6 ± 9.7, 60.6% male) who underwent coronary angiography; the sample was composed of 78 patients (mean age 60.2 ± 9.7, 70.5% male) with CSF and 92 patients (mean age 62.7 ± 9.5, 52.1% male) with normal coronary flow (NCF). As shown in Table 1, men were more prevalent in the CSF group than in the NCF group (70.5% versus 52.1%, *P* = 0.018). However, the other demographic factors, such as age, BMI, waist circumference, prevalence of hypertension, prevalence of diabetes and current smoking, did not differ significantly between the CSF and NCF groups. Except for TGs, TC, and high-density lipoprotein cholesterol (HDL-c), the two groups showed no significant differences in most laboratory tests. The levels of HDL-C and TC were lower in the CSF group than in the NCF group (1.15 ± 0.07 versus 1.49 ± 0.29, P<0.001; 4.32 ± 0.85 versus 4.76 ± 1.01, *P* = 0.003), and the TG level was significantly higher in the CSF group (1.97 ± 1.57 versus 1.51 ± 0.66, *P* = 0.020).

**Table 1:** Clinical characteristics of the study populations

Characteristics	Total (170)	NCF (92)	CSF (78)	*P* value
**Clinical Characteristics**				
Age (years)	61.6 ± 9.7	62.7 ± 9.5	60.2 ± 9.7	0.085
Sex(Male)	103(60.6%)	48(52.1%)	55(70.5%)	0.018
BMI(kg/m^2^)	25.3 ± 2.8	25.1 ± 2.9	25.5 ± 2.7	0.341
Waistline (cm)	88.8 ± 10.2	87.9 ± 9.5	89.8 ± 10.9	0.215
Diabetes mellitus, n (%)	37(21.8%)	19(20.7%)	18(23.1%)	0.713
Hypertension, n (%)	112(65.9%)	67(72.8%)	45(57.7%)	0.051
Smoking, n (%)	23(13.5%)	13(14.1%)	10(12.8%)	0.826
**Laboratory parameters**				
FBG (mmol/L)	6.28 ± 1.89	6.16 ± 1.82	6.41 ± 1.98	0.407
HbA1C (%)	2.95 ± 3.41	3.21 ± 3.43	2.65 ± 3.38	0.290
SBP (mmHg)	129 ± 15	128 ± 15	129 ± 15	0.688
DBP (mmHg)	76 ± 10	78 ± 10	76 ± 10	0.677
TG (mmol/L)	1.72 ± 1.19	1.51 ± 0.66	1.97 ± 1.57	0.020
TC (mmol/L)	4.56 ± 0.96	4.76 ± 1.01	4.32 ± 0.85	0.003
HDL-C (mmol/L)	1.33 ± 0.28	1.49 ± 0.29	1.15 ± 0.07	<0.001
LDL-C (mmol/L)	2.63 ± 0.77	2.73 ± 0.80	2.51 ± 0.71	0.064
Creatinine (μmol/L)	75.6 ± 24.1	75.3 ± 24.0	75.8 ± 24.2	0.870
Hs-CRP(mg/L)	2.44 ± 3.16	2.09 ± 3.00	2.84 ± 3.31	0.127
WBC (× 10^9^/L)	6.24 ± 1.63	6.12 ± 1.69	6.38 ± 1.56	0.287
Ejection fraction (%)	65.4 ± 6.4	66.1 ± 6.3	64.5 ± 6.6	0.103
Lp-PLA2 (ng/mL)	209.2 ± 88.6	141.9 ± 49.7	288.6 ± 50.3	<0.001
**Medication**				
Beta-blockers, n (%)	51(70%)	29(31.5%)	22(28.2%)	0.737
ACEI/ARB, n (%)	52(30.1%)	29(31.2%)	23(29.5%)	0.868
Statins, n (%)	66(38.8%)	33(35.9%)	33(42.8%)	0.432
Insulin, n (%)	62(36.5%)	36(39.1%)	26(33.3%)	0.523

*BMI* Body mass index, *FBG* Fasting blood glucose, *HbA1c* Glycosylated hemoglobin A1c, *SBP* Systolic blood pressure, *DBP* Diastolic blood pressure, *LDL-c* Low density lipoprotein cholesterol, *HDL-c* High density lipoprotein cholesterol, *TC* total cholesterol, *TG* triglyceride, *Hs-CRP* hyper-sensitive C-reactive protein, *WBC* white blood cell

**Fig. 1:**
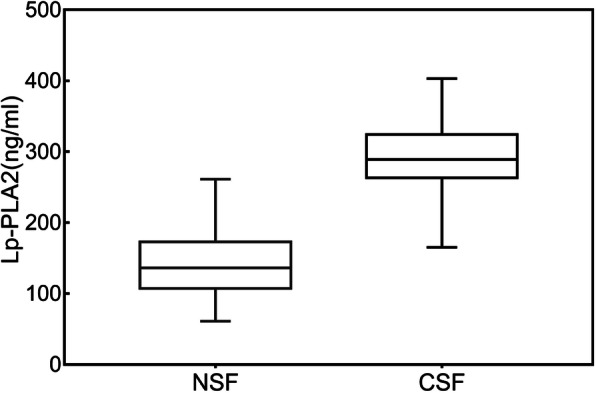
Lp-PLA_2_ expressed in the CSF group compared with the patients in the NCF, the difference was statistically significant. Lp-PLA_2_ lipoprotein-associated phospholipase A2, CSF coronary slow flow, NSF normal slow flow

**Fig. 2:**
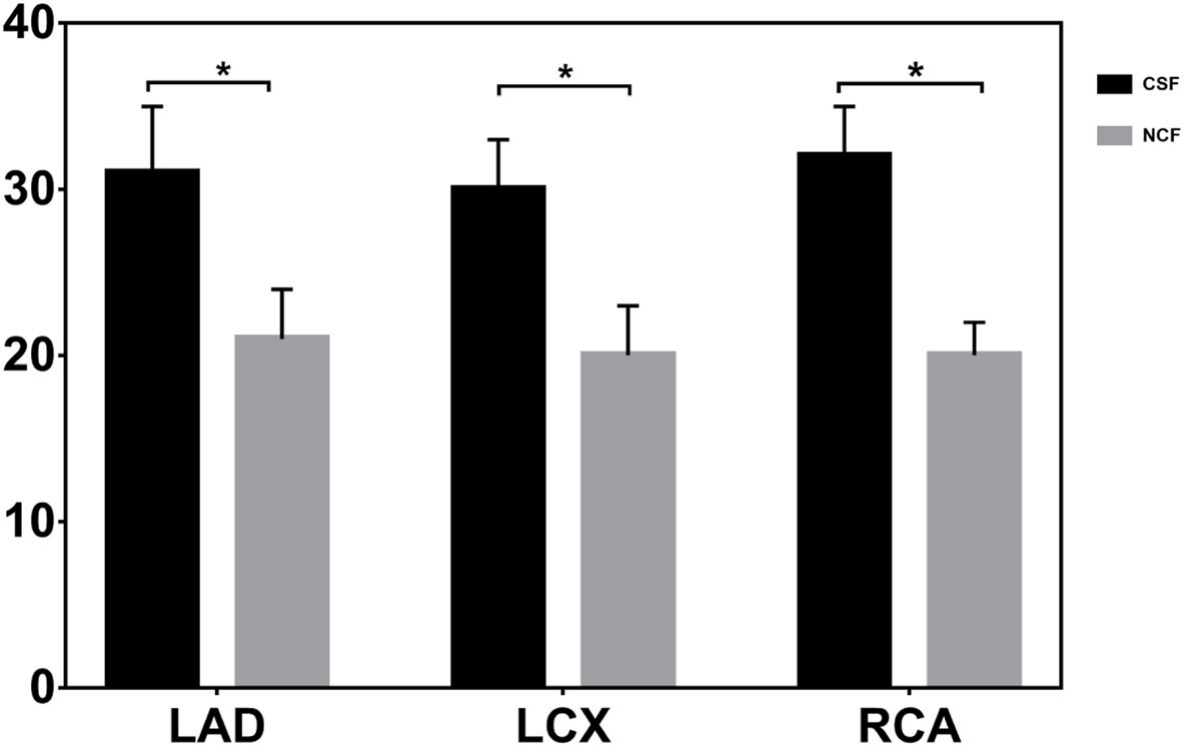
Angiographic bar graph for Subjects With Slow Flow and normal flow. LAD left anterior descending coronary artery, LCX left circumflex coronary artery, RCA right coronary artery, CSF coronary slow flow, NSF normal slow flow

Lp-PLA_2_ levels in the CSF group were significantly higher than those in the control group (288.6 ± 50.3 versus 141.9 ± 49.7, *P* < 0.001) (Table 1,Fig. 1). The TFC values of the two groups of coronary arteries were calculated respectively. The mean TFCs of LAD, LCX and RCA were significantly higher in CSF patients than in NCF patients (*p* < 0.001 for each coronary artery, Table 2, Fig. 2).

**Table 2:** Comparison of TIMI frame counts between patient groups with and without coronary slow flow phenomenon

Vessel	NCF (*n* = 92)	CSF (*n* = 78)	*P* value
LAD	21 ± 3	31 ± 4	<0.001
LCX	20 ± 3	30 ± 3	<0.001
RCA	20 ± 2	32 ± 3	<0.001
Mean	21 ± 2	31 ± 2	<0.001

*LCX* left circumflex coronary artery, *LAD* left anterior descending coronary artery, *RCA* right coronary artery, *TIMI* thrombolysis in myocardial infarction

**Fig. 3:**
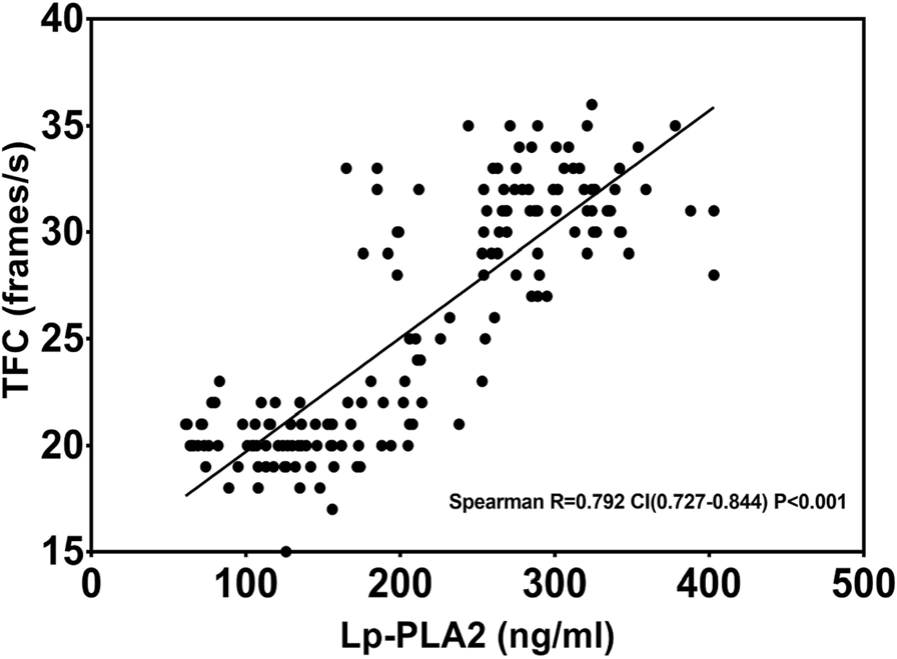
Spearman correlation analysis of Lp-PLA_2_ value with coronary artery TIMI frame count. Lp-PLA_2_ levels were significantly positively correlated with TFC. Lp-PLA_2_ lipoprotein-associated phospholipase A2, TFC thrombolysis in myocardial infarction frame count

**Fig. 4:**
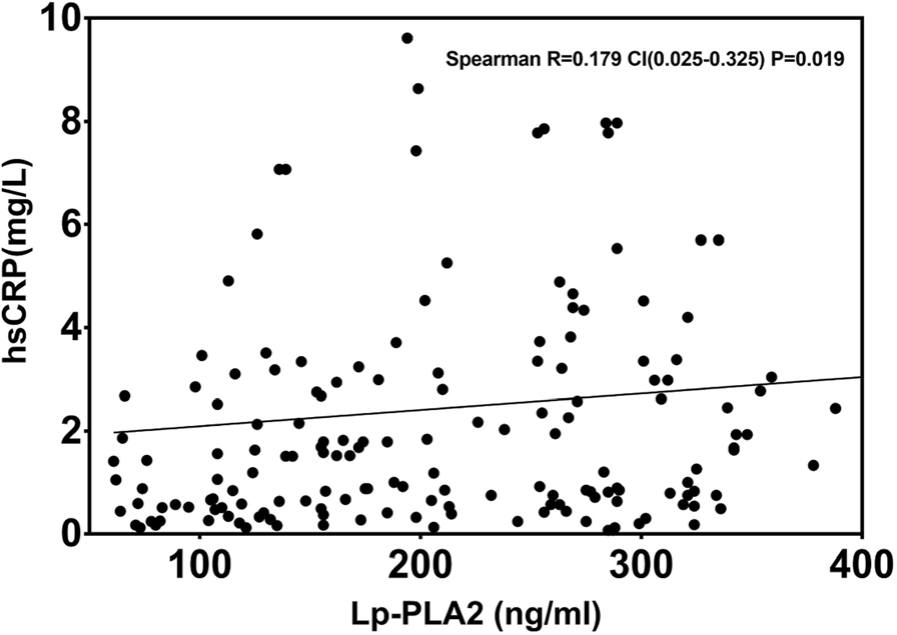
Spearman correlation analysis of Lp-PLA_2_ value with hs-CRP, Lp-PLA_2_ levels were significantly positively correlated with hs-CRP. Lp-PLA_2_ lipoprotein-associated phospholipase A2, hs-CRP

**Fig. 5:**
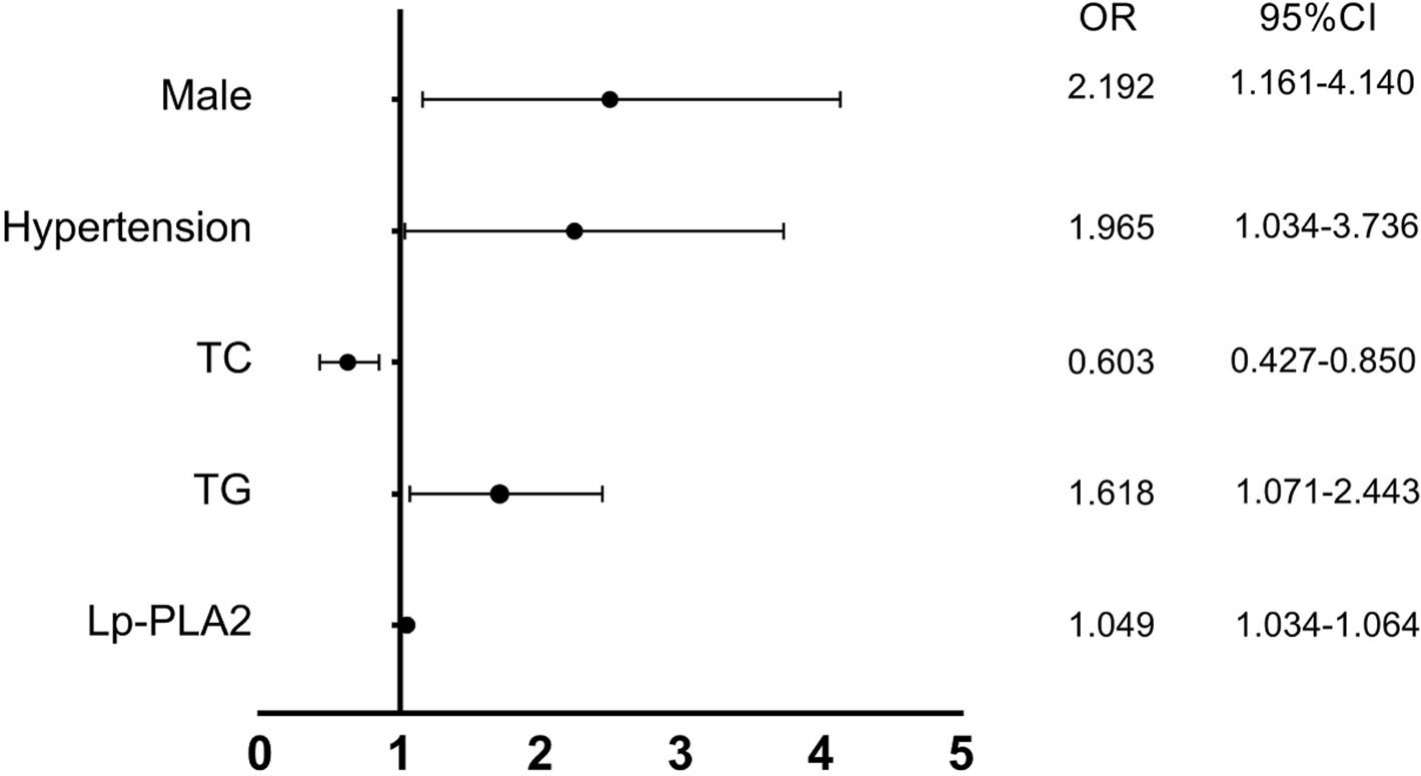
Multivariate logistic regression analysis of the risk of CSF. Lp-PLA_2_ lipoprotein-associated phospholipase A2, TC total cholesterol; TG

The spearman analysis, shown in Table 3, showed that the plasma Lp-PLA2 level was positively correlated with mean TFC (r = 0.790, P<0.001) and C-reactive protein (CRP), (r = 0.179, *P* = 0.019), and negatively correlated with HDL-C (r = − 0.693,P<0.001) and ejection fraction (r = − 0.164, *P* = 0.033) (Figs. 3, 4). Univariate and multivariate logistic regression analyses were used to explore the associations of risk factors with CSF. The results indicated that the level of Lp-PLA_2_ (OR = 1.049, CI = 1.034–1.064, *P* < 0.001), male sex (OR = 2.192, CI = 1.161–4.140, *P* = 0.016) and hypertension (OR = 1.965, CI = 1.034–3.736, *P* = 0.039) were risk factors for CSF. After adjustment for traditional confounders, we found that the Lp-PLA_2_ levels remained to be significantly and independently associated with the presence of CSF (OR = 1.040, CI = 1.022–1.059, *P*<0.001) (Table 4, Table 5, Fig. 5).

**Table 3:** Spearman’s correlation coefficients of Lp-PLA2 level with covariates

Variable	Correlation coefficient	*P* value
Age	−0.094	0.223
BMI	0.038	0.625
Waistline	0.087	0.261
FBG	0.122	0.114
HbA1C	−0.028	0.722
TG	0.277	<0.001
TC	−0.198	0.010
HDL-C	−0.693	<0.001
LDL-C	0.061	0.432
Creatinine	0.088	0.255
Hs-CRP	0.179	0.019
WBC	0.084	0.274
Ejection fraction	−0.164	0.033
TFC	0.790	<0.001

*BMI* Body mass index, *FBG* Fasting blood glucose, *HbA1c* Glycosylated hemoglobin A1c, *LDL-c* Low density lipoprotein cholesterol, *HDL-c* High density lipoprotein cholesterol, *TC* total cholesterol, *TG* triglyceride, *Hs-CRP* Hyper-sensitive C-reactive protein, *WBC* white blood cell

**Table 4:** Univariate regression analysis of the association of coronary slow flow with variables

Variables	OR	95% CI	*P* value
Age	0.972	0.942–1.004	0.087
Male	2.192	1.161–4.140	0.016
BMI	1.055	0.945–1.177	0.339
Waistline	1.019	0.989–1.050	0.216
Diabetes mellitus	1.153	0.556–2.391	0.703
Hypertension	1.965	1.034–3.736	0.039
Smoking	1.119	0.461–2.714	0.804
FBG	1.071	0.911–1.258	0.407
HbA1c	0.953	0.871–1.042	0.288
TC	0.603	0.427–0.850	0.004
TG	1.618	1.071–2.443	0.022
LDL	0.682	0.454–1.025	0.066
HDL	0.991	0.955–1.06	0.244
Creatinine	1.001	0.989–1.014	0.869
Hs-CRP	1.081	0.975–1.197	0.138
WBC	1.107	0.918–1.336	0.287
Ejection fraction	0.961	0.916–1.008	0.104
Lp-PLA_2_	1.049	1.034–1.064	<0.001

*BMI* Body mass index, *FBG* Fasting blood glucose, *HbA1c* Glycosylated hemoglobin A1c, *LDL-c* Low density lipoprotein cholesterol, *HDL-c* High density lipoprotein cholesterol, *TC* total cholesterol, *TG* triglyceride, *Hs-CRP* hyper-sensitive C-reactive protein, *WBC* white blood cell

**Table 5:** Multivariate logistic regression analysis of the risk of CSF with Lp-PLA_2_

	OR	95% CI	P
Model 1	1.031	1.022–1.040	<0.001
Model 2	1.041	1.022–1.061	<0.001
Model 3	1.040	1.022–1.059	<0.001

Odds ratio and 95% confidence intervals(CI) were obtained by the multivariate logistic regression model: model 1:After adjustment the age, gender, BMI, hypertension, Diabetes mellitus, smoking; model 2: model 1+ laboratory parameters in Fig. 1; model 3: model 2 + medicine

**Fig. 6:**
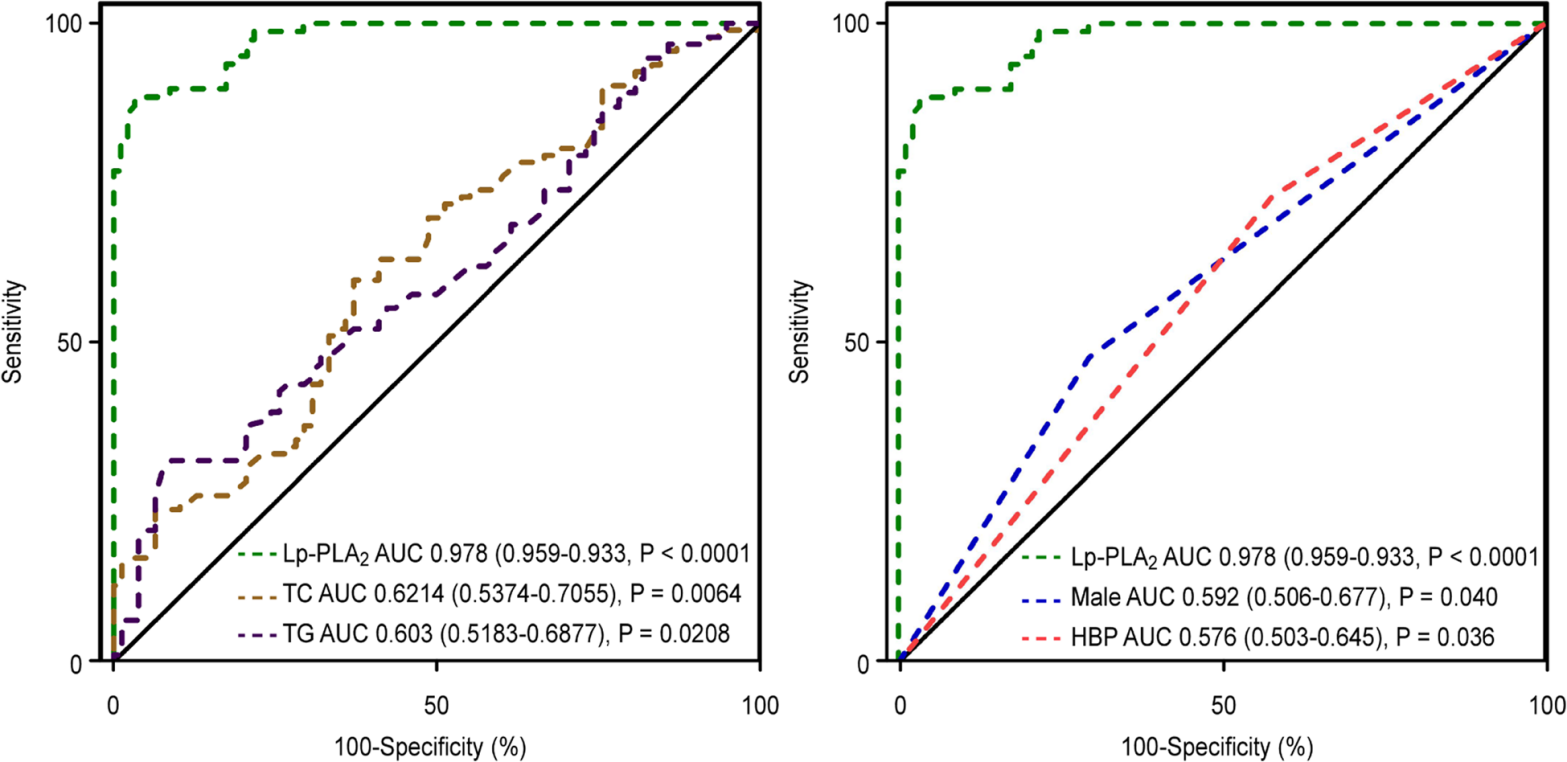
Receiver-operating characteristic curve analysis showing the prognostic value of Lp-PLA_2,_ TC, TGs, male sex, and hypertension. Lp-PLA_2_ lipoprotein-associated phospholipase A2, TC total cholesterol; TG triglyceride, HBP hypertension

The ROC curve analysis (Fig. 6) showed that Lp-PLA_2_ (AUC values = 0.978; CI = 0.959–0.993; *P* < 0.0001) was better in predicting CFS than TC (0.621; CI = 0.537–0.706; *P* = 0.0064), TG (0.603; CI = 0.518–0.688; *P* = 0.021), male sex(0.592; CI = 0.506–0.677; *P* = 0.040)and hypertension (0.576; CI = 0.503–0.645; *P* = 0.036). The sum of the sensitivity and specificity for the prediction of the extent of CSF was maximal at a level of Lp-PLA2 ≥ 260.5 ng/ml (sensitivity = 76.9% [95% CI 66 to 85.71%], specificity =98.9% [95% CI 94.1 to 99%]).

## Discussion

As far as we know, the present study demonstrated for the first time that there was an independent relationship between Lp-PLA_2_ and CSF phenomenon. The major finding was that Lp-PLA_2_ levels were positively correlated with CSF and significantly elevated in subjects with CSF. In addition, multivariate logistic regression analysis and ROC curves showed that Lp-PLA_2_ was still independently associated with the presence and severity of CSF. Male sex and hypertension also independently predicted the presence of the CSF phenomenon.

Although CSF has been found to be related to microvascular diseases, vascular endothelial dysfunction, coronary atherosclerosis, oxidative stress, insulin resistance, adipocytokines, and abnormal blood composition, the exact mechanisms are still unknown [18–20]. Tambe proposed that the CSF phenomenon might be related to abnormal microcirculation in 1972 [2]. Oğuzhan Çelik et al. identified a correlation between the extent of disruption in endothelial function and the CSF level using the flow-mediated dilation (FMD) method [21]. Several studies have found that the imbalance between endothelin-1 and nitric oxide in patients with CSF supports the involvement of endothelial dysfunction in CSF etiopathogenesis [22]. An intravascular ultrasound study demonstrated that diffuse coronary calcification was present in 88% of CSF patients, and statin therapy has been shown to improve coronary flow in CSF subjects [23]. Decreased fractional flow reserve in CSF patients has been demonstrated by intravascular ultrasound (IVUS) to result in increased resistance in the epicardial coronary artery due to diffuse atherosclerotic disease [24].

The role of chronic inflammation-mediated endothelial injury in CSF has been widely recognized. Muhammed Oylumlu et al. revealed that the platelet-to-lymphocyte ratio could be an important risk factor in patients with CSF [25]. Li et al. showed that plasma CRP and IL-6 as inflammatory markers were positively correlated with TIMI frame count in patients with CSF [26]. In our study, Lp-PLA_2_ was shown to be an independent predictor of CSF. Lp-PLA_2_, known as platelet-activating factor acetylhydrolase, is a calcium-independent serine lipase [10]. Lp-PLA_2_ is synthesized by macrophages and other inflammatory cells and circulates in the human blood mainly with LDL granules (80–85%) and less often with HDL [9]. Lp-PLA_2_ is involved in the oxidative modification of vascular wall LDL to generate oxidized phospholipids and oxidized non-esterified fatty acids, which can promote the development of vascular inflammation and atherosclerotic plaques [27]. Lp-PLA_2_ has been identified as an independent predictor of cardiovascular disease (CVD),and has been recommended as an adjunct to traditional risk assessment in patients with moderate and high 10-year risk of CVD as defined by Framingham risk scores [28]. In the REGARDS study, Lp-PLA_2_ activity was associated with coronary heart disease (CHD) risk over 5.3 years based on Cox proportional hazards regression [29]. Our study found that in the CSF group, with the increase in TFC, Lp-PLA2 levels exhibited an increasing trend, and there was a significant positive correlation between CSF and Lp-PLA_2_. Additionally multivariate logistic regression analysis showed that Lp-PLA_2_ could independently predict CSF. However, the mechanisms underlying the relationship between Lp-PLA_2_ and CSF are not entirely understood. The most reliable hypothesis seems to be that CSF is caused by increased microvascular resistance induced by endothelial dysfunction due to Lp-PLA_2_ mediated chronic inflammation.

Numerous studies have attempted to define the demographics, characteristics and independent predictors of patients with CSF. Arbey Y found that current smoking was associated with CSF [30]. Other studies have shown that BMI and hypertension were predictors of CSF phenomena [31]. Compared to other studies, our study did not find an association between BMI and CSF. Our study also shows that Lp-PLA_2_, male gender,c and hypertension are independent risk factor of the presence of CSF phenomena. Tsimikas found in his study that there was a significant correlation between Lp-PLA_2_ and LDL, non-HDL, and HDL [11]. However, we did not find any difference in LDL between the two groups, which may be related to the use of lipid-lowering drugs.

### Limitations

Our study has some limitations: (1)This study was limited by a small and nonrandomized sample, which may restrict the generalizability of our results. (2)The study did not examine the ethnic background or socioeconomic status of the study population.(3)This study was also a single center, retrospective study. Multicenter randomized controlled trials must be conducted to explore the relationship between Lp-PLA_2_ and CSF. (4)The use of drugs, such as lipid-lowering drugs, may affect the results of experiments. (5)Finally, we determined only the mass of Lp-PLA_2_ instead of its activity due to our laboratory limitations.

## Conclusion

In summary, the main finding was that Lp-PLA_2_ levels were positively correlated with CSF. The multivariate regression results showed that Lp-PLA_2_ was independently associated with the presence and severity of CSF. Currently, there is still no effective method to treat CSF. This study indicated the association between Lp-PLA_2_ and CSF, which may provide a new therapeutic target for the treatment of CSF. However, the exact pathophysiological mechanisms of Lp-PLA_2_ in CSF require further study to elucidate.

## Data Availability

The data and materials can be used with permission.

## Abbreviations

CSF: Coronary slow flow
Lp-PLA_2_: Lipoprotein-associated phospholipase A2
TFC: Thrombolysis in myocardial infarction frame count
RCA: Right coronary artery
LCX: Left circumflex coronary artery
LAD: Left anterior descending coronary artery
CVD: Cardiovascular disease
TC: Total cholesterol
TG: Triglyceride
HDL-C: High-density lipoprotein cholesterol
LDL-C: Low-density lipoprotein cholesterol
HbA1c: Glycosylated hemoglobin A1c
FBG: Fasting blood glucose
BMI: Body mass index
CRP: C-reactive protein
ROC: Receiver-operating characteristic curve
